# *Mycobacterium avium* Infection in a Domestic Shorthair Cat Following Subdermal Hyaluronic Acid Filler Injection

**DOI:** 10.1111/vop.70122

**Published:** 2025-12-01

**Authors:** Alexandra E. Bergen, Callie Miller, J. Seth Eaton, Lara M. Tomich, Jennifer L. Heyward, Taylor A. Opgenorth

**Affiliations:** ^1^ Department of Medical Sciences, School of Veterinary Medicine University of Wisconsin‐Madison Wisconsin Madison USA; ^2^ Department of Surgical Sciences, School of Veterinary Medicine University of Wisconsin‐Madison Wisconsin Madison USA

## Abstract

**Objective:**

To describe the diagnosis and treatment of a *Mycobacterium avium* ( *M. avium* ) infection in a cat following subdermal hyaluronic acid (HA) filler injection.

**Animal Studied:**

A five‐year‐old spayed female domestic shorthair cat with chronic inferior lateral entropion and chronic feline atopic skin syndrome (FASS).

**Procedures:**

Left inferior eyelid swelling and ipsilateral mandibular lymphadenomegaly developed approximately 2 weeks following subdermal HA injection OU. Culture of a fine needle aspirate (FNA) from the affected lymph node confirmed a *Mycobacterium avium* subsp. *hominissuis* infection resistant to most conventional antibiotics including fluoroquinolones and rifampin. Clinical response to sustained clarithromycin was poor and antitubercular isoniazid was poorly tolerated. As adjunctive therapy, two sequential intralesional injections with amikacin (12.5 mg/kg total dose per treatment) were performed into the affected eyelid and lymph node. Clinical reductions in eyelid swelling and lymphadenomegaly were observed thereafter. Approximately 4 months after diagnosis, mycobacterial PCR from an FNA of the affected lymph node was negative. Antibiotic therapy was completed approximately 6 months following diagnosis. No clinical signs of infection recurrence were present at the time of writing approximately 18 months following completion of antibiotic therapy.

**Conclusions:**

Non‐tuberculous mycobacterial (NTM) infections are a known and documented complication of subdermal filler injections in human patients. This is the first described case of NTM infection associated with HA injection in a cat. Treatment success with conventional oral antibiotics may be limited, necessitating extended therapeutic courses and alternative drug delivery routes like intralesional injection.

## 1 Introduction

Entropion, abnormal inversion of the eyelid(s), is a common ophthalmic diagnosis in dogs and cats. Heritable and breed‐associated factors may predispose to primary entropion in either species. Feline entropion more commonly develops as a secondary consequence of age‐related tissue changes such as skin laxity and orbital soft tissue atrophy with enophthalmia [1, 2]. Correspondingly, affected cats are often older. Feline secondary entropion may also be associated with generalized dermatologic disease and ocular surface disease associated with feline herpesvirus‐1 [2].

Regardless of the cause of entropion, definitive correction is indicated to reduce the associated trichiasis and mitigate the risks of chronic ocular surface irritation and discomfort, ulcerative keratitis, and corneal sequestrum. In cats, surgery is the standard approach to correction, often using a Hotz‐Celsus blepharoplasty, wedge resection, or combination thereof [2, 3]. However, the risks of general anesthesia and surgery are often greater in older cats, particularly those with metabolic, renal, or cardiovascular comorbidities. A successful non‐surgical technique using subdermal hyaluronate acid (HA) injections to reduce eyelid laxity and discourage inversion has been described [4]. While not intended to be definitively corrective, this alternative approach is well‐tolerated, long‐acting, and can be performed awake or with mild sedation avoiding anesthetic risks [4]. Moreover, this approach may be more affordable, particularly if the effect is sustained.

Subdermal HA injections are regularly performed in human patients, most commonly for elective cosmetic or augmentative purposes [5]. Reported immediate side effects are typically mild, characterized by local, transient pain and inflammation at the injection site [6]. However, more serious and adverse local effects may include severe immediate or delayed inflammatory reactions, vascular occlusion, and non‐tuberculous mycobacterial (NTM) infection [6, 7, 8]. Non‐tuberculous mycobacterial species are ubiquitous and often isolated from the respiratory tract and cutaneous surface in humans and animals, avian feces, and environmental sources like soil and water, including plumbing systems [9]. In one report describing a cluster of *Mycobacterium chelonae* infections in human patients following cosmetic filler injections, the same NTM species was cultured from the clinic tap water [10]. In many cases, however, a cause or predisposing factor for an NTM may be difficult to identify.

While NTM infection is a known risk in human patients receiving subdermal filler injections, the same complication has not been described in animals. The purpose of this case report is to describe an NTM infection following subdermal HA injection in a cat with entropion.

## 2 Case Report

### 2.1 Presenting Complaint and Diagnosis

A five‐year‐old female spayed domestic shorthair cat weighing 4.20 kg was presented to UW Veterinary Care (UWVC) with a 2‐week history of left inferior eyelid swelling and a firm left submandibular mass. The cat was a long‐term patient of the UWVC Dermatology and Ophthalmology Services with a chronic history of feline atopic skin syndrome (FASS) and inferior lateral entropion OU. Approximately 4 weeks prior to presentation, subdermal 2.4% hyaluronic acid injections (ALAYNA: an‐vision, West Jordan, UT, USA) had been routinely performed in both inferior eyelids. Following site preparation with 1.25% povidone iodine solution (prepared with sterile 0.9% sodium chloride solution) and sedation with intravenous (IV) dexmedetomidine (Dexmedesed; Dechra, Fort Worth, TX, USA) (10 μg/kg) and butorphanol (Zoetis, Parsippany, NJ, USA) (0.4 mg/kg), hyaluronic acid (HA) had been injected subdermally (0.3–0.4 mL OD, 0.1–0.2 mL OS) utilizing a published threading technique [4] with a 27‐gauge needle. Sterile gloves were worn by the injector. Sedation reversal had been performed with intramuscular (IM) atipamezole (Antisedan: Orion Corporation, Espoo, Finland, EU) (0.1 mg/kg) and recovery had been uneventful. Both doses of HA had been drawn from a newly unsealed manufacturer syringe, and sedatives and reversal agents were drawn from multidose vials stored in a password‐secured automated storage cabinet.

**FIGURE 1:**
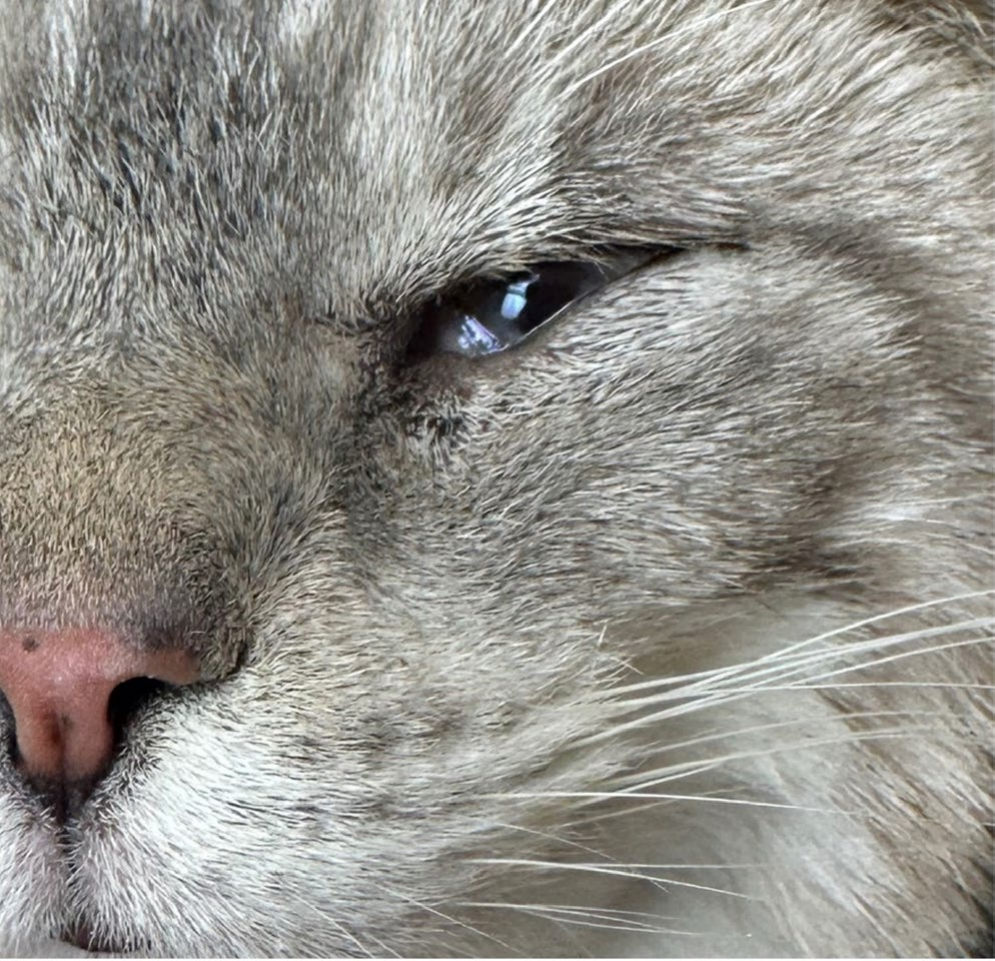
External photograph of the left eye approximately 4 weeks following subdermal hyaluronic acid injection of the inferior eyelid OS. No active entropion is present but induration and mild erythema of the eyelid skin are present.

At presentation, the left mandibular lymph node was moderately enlarged. The rest of the general physical examination was unremarkable. Ophthalmic examination comprising slit lamp biomicroscopy (SL‐17 portable slit lamp, Kowa American Corp, New York, NY, USA) and indirect ophthalmoscopy (Vantage Plus LED, Keeler, Malvern, PA, USA; 28D condensing lens, Volk Optical, Mentor, OH, USA) was performed. On slit lamp examination, no active entropion was present in either eye and both eyes were clinically comfortable. However, swelling and mild erythema were present adjacent to the left inferior eyelid margin and into the adjacent periocular skin (Figure 1). Corneal fibrosis and receding corneal neovascularization associated with previous entropion were observed inferotemporally OU. No other findings were observed on ophthalmic examination. At presentation, the cat was receiving topical erythromycin ophthalmic ointment (Bausch and Lomb Incorporated, Tampa, FL, USA) q8h OU and 30 mg (7 mg/kg) modified cyclosporine (Atopica, Elanco Animal Health, Greensboro, NC, USA) PO q48h for pre‐existing feline atopic skin syndrome.

**FIGURE 2:**
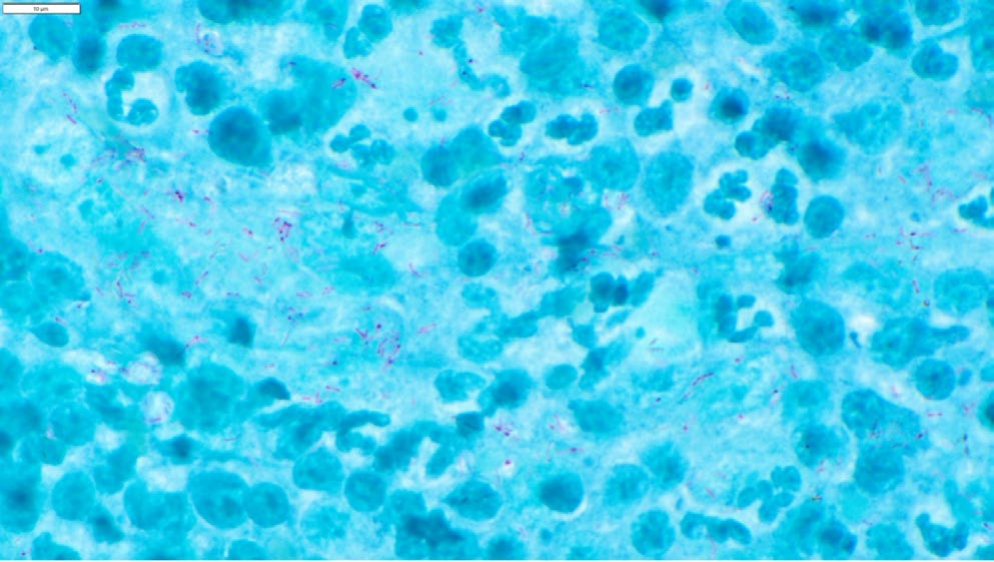
Photomicrograph (100×) of a fine needle aspiration sample from the enlarged left mandibular lymph node. Predominantly intracellular Kinyoun acid‐fast positive bacilli are seen. Scale bar at upper left indicates 10 μm. Photomicrograph courtesy of Allison Dusick, DVM, DACVP.

A sample from the affected lymph node was collected by fine needle aspiration (FNA) and submitted for cytologic evaluation. On cytology, predominantly lymphocytic inflammation was present with lesser populations of plasma cells, neutrophils, and epithelioid macrophages, and rare mast cells. Epithelioid macrophages were also present containing numerous short, linear, non‐staining bacilli consistent with a *Mycobacterium* species. The bacilli stained with moderate intensity using modified Kinyoun acid‐fast staining, supporting a clinical diagnosis of mycobacterial lymphadenitis (Figure 2). A second FNA was performed and submitted for mycobacterial culture and susceptibility testing (United States Department of Agriculture, National Veterinary Services Laboratories, Ames, IA, USA).

### 2.2 Initiation of Empirical Therapy

At diagnosis, oral cyclosporine (CsA) and ophthalmic erythromycin were discontinued. Pending culture results, empirical combination oral antibiotic therapy was prescribed according to published dose recommendations [11, 12, 13] and included 22.5 mg (5.4 mg/kg) pradofloxacin (Veraflox; Elanco, Greenfield, IN, USA) PO q24h, 32.5 mg (7.7 mg/kg) clarithromycin (Sandoz, Princeton, NJ, USA) PO q12h, and 25 mg (6 mg/kg) rifampin (Lupin, Naples, FL, USA) PO q12h. A twice‐daily dosing frequency for rifampin was chosen to mitigate the anticipated gastrointestinal side effects of the medication [11, 12]. Approximately 2 weeks after initiation of therapy, however, the cat was presented to UWVC for vomiting, lethargy, and inappetence. At that time, the left inferior eyelid swelling persisted and the mandibular lymph node remained enlarged. Due to the known hepatotoxic effects of rifampin, a serum liver panel was submitted and was normal. Based on an otherwise unremarkable assessment, the gastrointestinal signs and lethargy were attributed to the rifampin. The protocol was changed to 50 mg (12 mg/kg) PO q24h combined with an 8.5 mg (2 mg/kg) dose of maropitant (Cerenia; Zoetis, Parsippany, NJ, USA) given PO 1–2 h before dosing. Pradofloxacin and clarithromycin were continued as previously prescribed. According to owner report, signs of gastrointestinal upset subsided thereafter.

At recheck examination approximately 5 weeks after the change in rifampin dosing, eyelid swelling and lymphadenomegaly persisted and a recheck serum liver panel showed mildly increased values for AST (93 U/L [reference: 6–44]) and ALT (246 U/L [reference: 20–108]). Due to concern for early hepatotoxicity, rifampin was discontinued and pradofloxacin and clarithromycin continued as previously prescribed. At that time, the cat's owner also expressed concern about severe pruritus and self‐trauma associated with recurrent FASS. Therefore, CsA was resumed at the previous dose. A serum liver panel repeated 2 weeks later was normal. At that time, rifampin was resumed at once daily dosing (25 mg [6 mg/kg] PO q24h) and pradofloxacin and clarithromycin were continued.

### 2.3 Diagnosis and Definitive Treatment of *Mycobacterium avium* Complex (MAC) Infection

Approximately 10 weeks following initiation of empirical therapy, mycobacterial culture results were received confirming growth of *Mycobacterium avium* subsp. *hominissuis*. Susceptibility results (Table 1) demonstrated resistance of this *Mycobacterium avium* complex (MAC) isolate to most antibiotics including rifampin and two fluoroquinolones (ciprofloxacin and moxifloxacin), with only intermediate susceptibility to clarithromycin and amikacin. The organism was only categorized as susceptible to clofazimine, a riminophenazine antibiotic not readily available to veterinarians in the United States for off‐label treatment of individual animal patients. Due to the confirmation of a MAC infection, thoracic radiographs and abdominal ultrasound were performed with neither showing evidence of disseminated infection. Clarithromycin was continued at an increased dose (15 mg/kg PO q12h) [11, 13].

**TABLE 1:** Antimicrobial susceptibility patterns of the *Mycobacterium avium* subsp. *hominissuis* isolate cultured from lymph node aspirate.

Antimicrobial	Conc. tested (μg/mL)	MIC of isolate (μg/mL)	Interpretation
Amikacin	1–256	32	Intermediate
Ciprofloxacin	0.12–8	> 8	Resistant
Clarithromycin	0.06–64	4	Intermediate
Doxycycline	0.12–8	> 8	Resistant
Minocycline	0.06–8	> 8	Resistant
Clofazimine	0.015–4	0.25	Susceptible
Linezolid	1–32	32	Resistant
Moxifloxacin	0.015–4	4	Resistant
Rifabutin	0.12–4	1	Intermediate
Rifampin	0.004–4	> 4	Resistant
Streptomycin	0.5–32	> 32	Resistant
Trimethoprim‐Sulfa	0.25/4.75–4/76	> 4/76	Resistant

Abbreviation: MIC, minimum inhibitory concentration.

Due to the marked antibiotic resistance of the MAC isolate, consultation was sought with a recognized expert in veterinary infectious disease (Dr. Richard Malik) to explore therapeutic options. In light of the cat's poor clinical response to empirical systemic antibiotics, intralesional therapy with amikacin was recommended to augment local antibiotic concentration while avoiding the drug's potential nephrotoxic effects. Antitubercular medications like ethambutol and isoniazid were also discussed as possible alternative therapies by the consulting regulatory laboratory (United States Department of Agriculture, National Veterinary Services Laboratories, Ames, IA, USA). However, given the known toxic profiles of these drugs, DNA sequencing of the isolate for genetic resistance to each was first recommended to verify susceptibility.

Approximately 2 weeks after culture results were received, intralesional amikacin injections were performed as outlined in Table 2. Prior to treatment, a serum biochemistry panel was submitted and all values were normal. Additionally, 100 mL of 0.9% NaCl were administered subcutaneously over the dorsum to ensure hydration and mitigate the potential for aminoglycoside‐associated renal toxicity. Sedation was administered with IV dexmedetomidine, the skin overlying the injection sites clipped and aseptically prepared, and subdermal and direct intranodal injections administered into the left inferior eyelid and mandibular lymph node, respectively. Sedation was reversed with IM atipamezole and recovery was uneventful. Following injection, oral clarithromycin was continued as previously prescribed. Four days after the injections, serum BUN and creatinine and urine specific gravity (USG) were measured and were within normal limits.

**TABLE 2:** Intralesional amikacin injection procedures.

1. Serum BUN and creatinine were measured pre‐emptively
2. 100 mL 0.9% NaCl were administered subcutaneously over the dorsum
3. Dexmedetomidine (4 μg/kg) was administered IV
4. Injection sites were clipped and prepped with 1.25% povidone iodine solution
5. Intralesional injections were performed^a^
a. 25 mg (6.25 mg/kg) subcutaneously, left inferior eyelid and adjacent skin
b. 25 mg (6.25 mg/kg) intranodal, left mandibular lymph node
6. Sedation was reversed with 1 mg/kg atipamezole IM
7. 4 days post‐injection, serum BUN and urine specific gravity were measured to monitor for adverse renal effects

^a^ Injection was performed using a 25‐gauge needle.

A second amikacin injection was scheduled approximately 6 weeks following the first. In the interim, results of isolate DNA sequencing were received revealing the presence of resistance genes to ethambutol but absence of resistance genes to isoniazid. Isoniazid (Teva, Parsippany, NJ, USA) was added to clarithromycin treatment at 50 mg (12.5 mg/kg) PO q24h. However, isoniazid was discontinued after 10 days due to reported vomiting, lethargy, and inappetence.

At presentation for the second amikacin injection, eyelid swelling and lymphadenomegaly persisted but were subjectively improved. A sample from the lymph node was collected by FNA and submitted for mycobacterial PCR and was negative. A second intralesional amikacin injection was performed in the same manner as previously described.

Shortly after the diagnosis of MAC infection, a syringe of HA from the same manufacturer lot of the syringe used at the initial subdermal injection was submitted for mycobacterial culture and was negative. Quantitative mycobacterial PCR (Environmental Safety Technologies, Louisville, KY, USA) of water from the clinic sink where clinicians frequently wash their hands was also performed to screen for the presence of viable *M. avium* organisms and was also negative.

Clarithromycin treatment was continued for 2 additional months. At completion of therapy approximately 6 months following diagnosis, swelling of the left eyelid and mandibular lymph node enlargement had resolved.

## 3 Follow‐Up to Date

Approximately 7 months following treatment completion, no eyelid swelling or lymph node enlargement was observed but inferior lateral entropion had recurred OS. At that time, the owner elected for surgical correction which was performed using a routine combined Hotz‐Celsus and wedge resection blepharoplasty. During surgery a segment of the resected inferior eyelid skin was submitted for mycobacterial PCR and was negative. At the time of writing approximately 18 months after completion of antibiotic therapy, the cat remains healthy with no recurrence of entropion and no eyelid or lymph node swelling.

## 4 Discussion

This case report is the first published description of a non‐tuberculous mycobacterial infection following subdermal HA injection for treatment of entropion in a cat. It is possible that the MAC infection may have been unrelated to the injection, but this is considered implausible for several reasons. Foremost, eyelid swelling and ipsilateral lymphadenitis developed concurrently, and both persisted for similar durations and ultimately responded similarly to treatment. Furthermore, the infection developed approximately 2–4 weeks following injection, a delayed interval typical of slow‐growing mycobacterial species like *M. avium* following cutaneous inoculation [14]. Demonstration of mycobacterial organisms in FNA samples from the left eyelid at diagnosis would have further supported the diagnosis but was not performed.

Feline NTM infections are predominantly localized to the cutaneous tissues; however, MAC‐associated infections may be more likely to disseminate than other non‐tuberculous isolates [15, 16, 17, 18]. In this cat disseminated infection was not identified. Moreover, the cat lacked a history of known environmental risk factors (outdoor lifestyle, exposure to other cats or birds) and was relatively young and free of systemic comorbidities that could be considered predispoing [19, 20]. However, immunocompromise is a known risk factor for NTM infection in human patients and may be a predisposing factor in cats [21, 22, 23, 24, 25]. Though the dose of CsA prescribed for FASS control in this cat at the time of infection was low in comparison to a prior report [24], it is plausible that iatrogenic immunocompromise was a predisposing factor for local infection after percutaneous inoculation [25].

Complementary diagnostic efforts in this case were focused on identifying potential industrial sources associated with the HA injection procedure. However, culture of the origin lot of the injected HA and PCR of the clinic tap were negative. Based on human evidence [26], it is possible that a swab from within the tap itself or culture of a water sample may have been more likely to identify an *M. avium* species. Without an identifiable environmental source or verifiable contamination of the HA lot, and use of iodine preparation and sterile gloves at HA injection, cutaneous contamination of the needle is deemed the most plausible cause. It is noteworthy that mycobacterial species, including strains of *M. avium* , are frequently less susceptible to povidone iodine than non‐mycobacterial microbes [27]. Thus, contamination of the skin and/or needle at injection may occur even in the face of careful site preparation.

Diagnosis of NTM infections is a clinical challenge. Mycobacterial culture is considered the gold standard for identifying active infections and determining susceptibility. However, the fastidious growth requirements of NTM isolates in culture may also lead to false negative results [12]. Moreover, the slow‐growing nature of many MAC organisms frequently delays results which can complicate the initiation of effective treatment. In this case, culture and susceptibility results were not received until approximately 10 weeks after submission, necessitating an extended period of sub‐therapeutic empirical antibiotic treatment.

Unlike culture, mycobacterial PCR produces faster results than culture and is considered a standard clinical diagnostic test for identifying mycobacterial infections in veterinary species [28]. However, PCR specificity and sensitivity may be reduced by non‐viable mycobacterial DNA unassociated with active infection and low bacterial load, respectively. These shortcomings can limit the utility of PCR in monitoring active mycobacterial infections and response to therapy. Therefore, simultaneous submission of mycobacterial culture and PCR is advocated; however, this incurs greater cost and longer turnaround time and can be less practical in the veterinary clinic [12]. Acknowledging these limitations, PCR was chosen for monitoring this cat and was interpreted in the context of clinical indicators of treatment response. It is noteworthy that culture was specifically chosen for testing the HA syringe as glycosaminoglycans may interfere with nucleic acid extraction and amplification required for PCR [29].

Mycobacterial organisms have uniquely thick cell walls, are slow‐growing, and possess other intrinsic resistance mechanisms. As a result, the identification of effective and well‐tolerated treatment options can be complicated and may differ between human and veterinary patients (Data S1). In most cases, extended courses of combination antimicrobial therapy are necessary, often lasting at least 6 months as seen in this case [12, 19, 23]. Additionally, the frequent need for multiple antibiotics and antitubercular drugs may be associated with poor tolerability and side effects such as nephro‐ and hepatotoxicity, limiting their efficacy [30, 31]. Poor response and tolerability of oral therapy in this cat necessitated an alternative approach using intralesional therapy. While this approach is not routinely recommended or considered standard by physicians, it has been described in human patients with cutaneous NTM infections following filler injections [32].

Due to the short half‐life of amikacin in subcutaneous tissue [33], more frequent amikacin injections would have been preferred to provide sustained delivery. However, our dosing approach was limited by the practical disadvantages of frequent sedation and the need for intensive monitoring for toxicity as well as owner convenience and cost considerations. Despite the long intervals between treatment, intralesional injections in this cat were well‐tolerated and appeared to expedite clinical improvement as an adjunct to an extended course of oral antibiotic.

In conclusion, this case represents the first documented instance of NTM infection in a cat following subdermal HA injection for entropion. Moreover, it is the first detailed description of any adverse complication associated with this approach for the management of feline entropion. The authors emphasize, however, that this procedure should still be regarded as low‐risk in cats as the consequence presented here is believed to be rare. This case also provides valuable insights into the diagnostic and therapeutic dilemmas encountered in cats with NTM infections, including antibiotic resistance and poor tolerability of traditional therapy. The clinical challenges encountered here underscore the need for close monitoring and careful physical examination post‐injection, particularly in cats with known environmental or patient‐related risk factors.

## Author Contributions

**Alexandra E. Bergen:** writing – original draft, methodology, writing – review and editing, formal analysis, data curation. **Callie Miller:** conceptualization, investigation, writing – original draft, writing – review and editing, formal analysis, data curation. **J. Seth Eaton:** conceptualization, investigation, writing – original draft, methodology, writing – review and editing, formal analysis, project administration, data curation. **Lara M. Tomich:** conceptualization, investigation, writing – original draft, writing – review and editing, formal analysis, data curation. **Jennifer L. Heyward:** conceptualization, investigation, writing – original draft, writing – review and editing, formal analysis, data curation. **Taylor A. Opgenorth:** conceptualization, investigation, writing – original draft, writing – review and editing, formal analysis, data curation.

## Disclosure

The authors have not used AI to generate any part of this manuscript.

## Conflicts of Interest

The authors declare no conflicts of interest.

## Supporting information


**Data S1:** Supplementary Clinical commentary.

## Data Availability

The data that support the findings of this study are available from the corresponding author upon reasonable request.
