# Supplementary material for: Mycobacterium avium Infection in a Domestic Shorthair Cat Following Subdermal Hyaluronic Acid Filler Injection
Source: Vet Ophthalmol. 2025 Dec 1;29(2):e70122. doi: 10.1111/vop.70122 (PMC12963522; doi:10.1111/vop.70122)
Supplement: Supplementary file 1 — Data S1: Supplementary Clinical commentary. [file VOP-29-0-s001.docx]

**Supplementary Commentary**

# This commentary provides detailed rationale for some of the clinical microbiological and therapeutic decisions made in managing this feline case of *Mycobacterium avium* complex (MAC) soft tissue infection. Specifically, it highlights key comparative aspects of the clinical approaches to non-tuberculous mycobacterial (NTM) in human and veterinary medicine, clarifying important differences and providing context for challenges and considerations that were necessary for treating this animal patient. **Antimicrobial Selection Strategy**

In human patients, minimum inhibitory concentrations (MIC) for ethambutol and rifampin have not been shown to correlate with clinical outcomes when treating Mycobacterial infections.(1) Therefore, categorization of resistance for each based on MIC may be flawed, and both drugs are often used regardless of MIC. Clinical outcome has only been correlated with macrolide antibiotics and amikacin.(2)

Extrapolation of correlative data from human to animal patients is complicated by numerous translational factors; and treatment guidance using MICs is still considered the standard of practice in veterinary medicine. Antimicrobial choices in this case were therefore chosen balancing MIC in tandem with other clinical considerations like tolerability, practicality, and availability. The presence of resistance genes to ethambutol led these authors to seek other options, particularly given its poor tolerability profile in cats.(3) Amikacin was not considered a practical first-line option in our patient due to the need for regular parenteral doses as well as the risk of renal toxicity, particularly in a cat concurrently receiving oral cyclosporine.

Empirical antimicrobial therapy prior to definitive isolate identification was initiated using a recommended broad-spectrum combination of antibiotics that included clarithromycin, rifampin, and a fluoroquinolone.(3–5) Pradofloxacin was included based on its reported in vitro activity against rapidly growing mycobacteria in feline patients(4), although its efficacy against MAC specifically is limited. Its inclusion was justified by the need for early intervention using a fluoroquinolone with proven safety in comparison to other fluoroquinolones in cats((6).

Clofazimine was considered as a potential adjunctive therapy. However, its use in veterinary medicine is restricted due to regulatory limitations. Access requires an Investigational New Animal Drug (INAD) application, which was not feasible for this case given the time-sensitive nature of treatment. Furthermore, permission for INAD use in individual animal patients appears to most directly apply to larger veterinary clinical trials or for serious or life-threatening conditions.(7)

**Diagnostic and Laboratory Considerations**

Susceptibility testing was performed at the USDA National Veterinary Services Laboratories (NVSL) using Clinical and Laboratory Standards Institute (CLSI) VET01 guidelines(8), which are focused on veterinary pathogens. Resistance gene testing for ethambutol and isoniazid was also conducted at NVSL to explore potential alternative therapies, although neither drug was ultimately used due to resistance findings and lack of veterinary precedent.

**Alternative Therapies**

Phage therapy has been described as an alternative approach to treatment of Mycobacterial infections in human patients and for treatment of various bacterial infections in animal patients(9–11). In this case, phage therapy was not pursued due to its limited availability and lack of established protocols for treatment of Mycobacterial infections in animals.

**References**

1. Moon SM, Kim SY, Kim DH, Huh HJ, Lee NY, Jhun BW. Relationship between Resistance to Ethambutol and Rifampin and Clinical Outcomes in Mycobacterium avium Complex Pulmonary Disease. Antimicrobial Agents and Chemotherapy. 2022 Mar 10;66(4):e02027-21.

2. Daley CL, Iaccarino JM, Lange C, Cambau E, Wallace RJ Jr, Andrejak C, et al. Treatment of Nontuberculous Mycobacterial Pulmonary Disease: An Official ATS/ERS/ESCMID/IDSA Clinical Practice Guideline. Clin Infect Dis. 2020 Aug 14;71(4):e1–36.

3. O’Halloran C, Gunn-Moore D. Mycobacteria in cats: an update. In Practice. 2017;39(9):399–406.

4. Govendir M, Norris JM, Hansen T, Wigney DI, Muscatello G, Trott DJ, et al. Susceptibility of rapidly growing mycobacteria and Nocardia isolates from cats and dogs to pradofloxacin. Veterinary Microbiology. 2011 Dec 15;153(3):240–5.

5. Lloret A, Hartmann K, Pennisi MG, Gruffydd-Jones T, Addie D, Belák S, et al. Mycobacterioses in Cats: ABCD guidelines on prevention and management. Journal of Feline Medicine and Surgery. 2013 Jul 1;15(7):591–7.

6. Messias A, Gekeler F, Wegener A, Dietz K, Kohler K, Zrenner E. Retinal safety of a new fluoroquinolone, pradofloxacin, in cats: assessment with electroretinography. Doc Ophthalmol. 2008 May 1;116(3):177–91.

7. Medicine C for V. How to Get a New Animal Drug Approved and First Steps to Get Started. FDA [Internet]. 2025 Apr 14 [cited 2025 Nov 9]; Available from: https://www.fda.gov/animal-veterinary/resources-you/how-get-new-animal-drug-approved-and-first-steps-get-started

8. Watts JL, Sweeney MT, Lubbers BV. Antimicrobial Susceptibility Testing of Bacteria of Veterinary Origin. Microbiology Spectrum. 2018 Mar 29;6(2):10.1128/microbiolspec.arba-0001–2017.

9. Dedrick RM, Smith BE, Cristinziano M, Freeman KG, Jacobs-Sera D, Belessis Y, et al. Phage Therapy of Mycobacterium Infections: Compassionate Use of Phages in 20 Patients With Drug-Resistant Mycobacterial Disease. Clin Infect Dis. 2023 Jan 1;76(1):103–12.

10. Köhne M, Hüsch R, Peh E, Hirnet J, Tönissen A, Müsken M, et al. Newly isolated bacteriophages show efficacy and phage-antibiotic synergy in vitro against the equine genital pathogens Klebsiella pneumoniae and Pseudomonas aeruginosa. BMC Vet Res. 2025 Oct 3;21(1):568.

11. Loponte R, Pagnini U, Iovane G, Pisanelli G. Phage Therapy in Veterinary Medicine. Antibiotics. 2021 Apr;10(4):421.

12. Malik R, Gabor L, Martin P, Mitchell D, Dawson D. Subcutaneous granuloma caused by Mycobacterium avium complex infection in a cat. Australian Veterinary Journal. 1998;76(9):604–7.
